# DRGquant: A new modular AI-based pipeline for 3D analysis of the DRG

**DOI:** 10.1016/j.jneumeth.2022.109497

**Published:** 2022-02-16

**Authors:** Matthew A. Hunt, Harald Lund, Lauriane Delay, Gilson Goncalves Dos Santos, Albert Pham, Zerina Kurtovic, Aditya Telang, Adam Lee, Akhil Parvathaneni, Emily Kussick, Maripat Corr, Tony L. Yaksh

**Affiliations:** a Departments of Anesthesiology and Pharmacology, University of California, San Diego, 9500 Gilman Drive, La Jolla, CA 92093, USA; b Department of Physiology, Karolinska Institutet, Stockholm, Sweden; c Department of Medicine, University of California, San Diego, 9500 Gilman Dr., La Jolla, CA 92093, USA; d Departments of Anesthesiology and Pharmacology, University of California, San Diego, 9500 Gilman Drive, La Jolla, CA 92093, USA

**Keywords:** Three dimensional (3D) analysis, Artificial intelligence, Whole mount, Dorsal root ganglion, Macrophage

## Abstract

**Background:**

The dorsal root ganglion (DRG) is structurally complex and pivotal to systems processing nociception. Whole mount analysis allows examination of intricate microarchitectural and cellular relationships of the DRG in three-dimensional (3D) space.

**New method:**

We present *DRGquant* a set of tools and techniques optimized as a pipeline for automated image analysis and reconstruction of cells/structures within the DRG. We have developed an open source software pipeline that utilizes machine learning to identify substructures within the DRG and reliably classify and quantify them.

**Results:**

Our methods were sufficiently sensitive to isolate, analyze, and classify individual DRG substructures including macrophages. The activation of macrophages was visualized and quantified in the DRG following intrathecal injection of lipopolysaccharide, and in a model of chemotherapy induced peripheral neuropathy. The percent volume of infiltrating macrophages was similar to a commercial source in quantification. Circulating fluorescent dextran was visualized within DRG macrophages using whole mount preparations, which enabled 3D reconstruction of the DRG and *DRGquant* demonstrated subcellular spatial resolution within individual macrophages.

**Comparison with existing method(s):**

Here we describe a reliable and efficient methodologic pipeline to prepare cleared and whole mount DRG tissue. *DRGquant* allows automated image analysis without tedious manual gating to reduce bias. The quantitation of DRG macrophages was superior to commercial solutions.

**Conclusions:**

Using machine learning to separate signal from noise and identify individual cells, DRGquant enabled us to isolate individual structures or areas of interest within the DRG for a more granular and fine-tuned analysis. Using these 3D techniques, we are better able to appreciate the biology of the DRG under experimental inflammatory conditions.

## Graphical Abstract

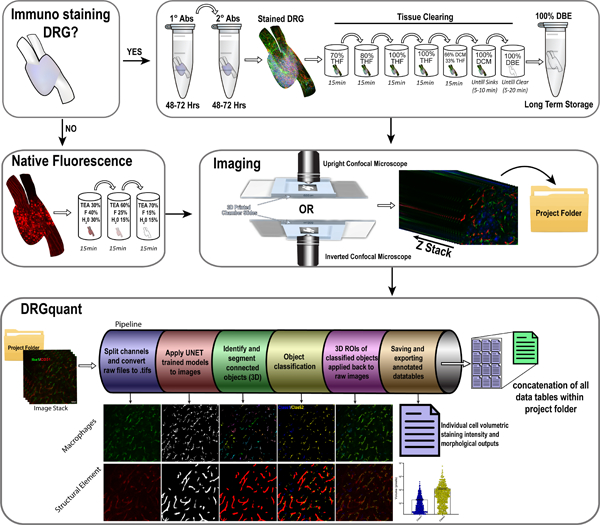

## 1. Introduction

The dorsal root ganglion (DRG) represents a complex niche environment, the importance of which has yet to be fully explored. In the DRG, neuronal soma, glomeruli, axons of passage, post ganglionic sympathetic axons, satellite glial cells, macrophages, and blood vessels are densely packed into a small niche environment, which lacks archetypical or laminar organization (Jimenez-Andrade et al., 2008). While DRG neuronal soma are spherical structures typically ranging in diameter from 15 μm to 50 μm (Ruscheweyh et al., 2007), other cell types, such as macrophages and satellite glia have more complex morphological features, which are integral aspects of their function.

Accordingly, given this morphological complexity, there is a high likelihood that any given thin section will cut through multiple portions of each of these ganglionic components. As the DRG lacks any lamination or nuclear organization by which the tissue may be oriented, this heterogeneous array requires multiple sections to be analyzed to achieve a meaningful dataset. Further, typical thin histological sections are suboptimal for defining the relationships among the complex cellular and structural elements of the DRG, as well as spatial niches including the neuronal soma rich region of the DRG (NSRR) and the nerve fiber rich region (FRR).

In our work, we have focused on DRG macrophages, which have been implicated as important mediators of pain states occurring after chronic inflammation and nerve injury (Yu et al., 2020). Macrophages are morphologically complex cells that extend filopodia in response to environmental signals in any and all directions (Horsthemke et al., 2017; Hu et al., 2019). Accordingly, when analyzing stained and sectioned DRGs, a macrophage may appear in and out of the plane of the section, making it difficult to accurately enumerate the number of macrophages, trace their complex morphology and define their physical proximity to other structural elements within the DRG. One approach to address this is to homogenize the tissue and isolate macrophages for flow cytometry. However, in doing so, we lose the cellular morphology and contextual information (e.g. arrangement within the DRG) that are of interest in identifying sub populations based on size, or types of cells that are in contact with a given macrophage. Such an analysis has assumed increasing importance given the proposed role of these resident cells in inflammatory and neuropathic pain states (Yu et al., 2020). Thus, we and others have, concluded that studying the DRG would benefit from whole mount immunohistochemistry (Bernal et al., 2019). As the DRG is typically under 400 μm in thickness in mice and under 600 μm in thickness in rat, it represents a uniquely ideal niche tissue suitable for fast and efficient whole mount tissue staining, clearing, and optical sectioning. As such we set out to find an improved methodology for studying macrophages in-situ in the DRG and ended up creating a platform and pipeline not only suitable for studying DRG macrophages, but which can be readily extended to any cell type or structures in the DRG (see Fig. S4 for example with satellite glial cells).

We first set out to optimize protocols for imaging endogenous fluorescence, as well as immunostained DRGs. Our goal was to establish protocols that took the same amount time as traditional tissue sectioning and histology. We then set out to establish an open access modular pipeline for automated image analysis, which we named *DRGquant.* In the examples provided here, *DRGquant*’s pipeline uses the power of modern GPUs and machine learning to aid in segmentation of macrophages and structural elements, as well as classification of individual cells based on their location within the DRG and quantifies each cell’s volume information, intensity of fluorophores, as well as a host of shape and morphological characteristics.

Here we present the methodology employed for DRG wholemount and then introduce *DRGquant,* which enables analysis and classification of macrophages. In order to characterize the power of this technique, we then present three important proof of principle experiments in which we ask whether we could (i) detect changes in DRG macrophage morphology or number in a model of innate immune mediated nociception, e.g. those states initiated by intrathecal lipopolysaccharide (LPS), or by chemotherapeutics leading to a polyneuropathy (e.g chemotherapy induced peripheral neuropath: CIPN); (ii) identify sub-populations of DRG macrophages; and (iii) assess DRG macrophages ability to uptake circulating fluorescently labeled agents.

## 2. Materials and methods

### 2.1. Animals

All animal experiments were carried out according to protocols approved by the Institutional Animal Care and Use Committee of UC San Diego and in concordance with the ARRIVE guidelines. Mice were housed with up to 4 litter mates per cage, and given food and water ad libitum, in a temperature-controlled facility with 12-hour light dark cycles. Wild type male C57BL/6 and Ccr*2*^rfp/rfp^ mice (B6.129(Cg)-Ccr2^tm2.1Ifc^/J; stock017586) were purchased from The Jackson Laboratory (Bar Harbor, ME) and bred and maintained at the University of California, San Diego. In this strain, a monomeric red fluorescent protein (RFP) sequence replaces the coding sequence of the *Ccr2* gene, abolishing gene function, hence they are here referred as *Ccr2*^−/−^.

### 2.2. Intrathecal drug delivery

Mice were anaesthetized with 2–3% Isoflurane, and the respiratory rate was monitored via observation. Stock LPS (50 μL aliquots at 2 mg/ml stored in −20°) was diluted (10 μL stock/ 90 μL saline) to a final concentration of 0.2 μg/μL, and Saline (NaCl 0.9%) for controls. AAV9-mCherry was generously donated by Prashant Mali (University of California, San Diego) and was delivered at 1xe^12^ total viral particles. Injections were performed as follows: A 25 μL Hamilton syringe affixed to a 30 g needle via polyethylene tubing PE10 was first flushed with sterile water and then loaded with 5 μL of LPS or saline. The 30 g needle was then inserted between the lumbar 4 (L4) and lumbar 5 (L5) intervertebral-space until a tail twitch was observed. The injection was performed slowly, during which, any compression of the air bubble within the tubing was be observed and noted as an indication of impeded flow.

### 2.3. Chemotherapy induced peripheral neuropathy (CIPN)

CIPN was induced using cisplatin (CIS; 2. 3 mg/kg/day; n = 15) (Spectrum Chemical MFG., Gardena, CA, USA) given intraperitoneally two times on Day 1 and Day 3. Saline (SAL; 0. 9% NaCl; n = 5) was injected in control mice. DRGs were collected at Day 10 as described below.

### 2.4. Intravenous dextran

Mice were deeply anesthetized with 2–4% isoflurane and transferred into a restraining device. The tail was cleaned with 70% ethanol and immersed in 38 °C water. The tail vein was visually identified and a 29 ½ gauge insulin syringe was used to administer 100 μL of 75 kDa Dextran, Tetramethylrhodamine (Invitrogen, #D1818). Mice were euthanized and perfusion fixed 1 h after injections.

### 2.5. Tissue collection

Mice were deeply anesthetized with isoflurane, and then euthanized with 0.1 ml of Beuthenasia (Merck) delivered into the intraperitoneal space. Mice were then transcardially perfused with ice cold saline followed by 4% PFA and post-fixed in 4% PFA for 24 h, then stored in PBS with 0.02% sodium azide. DRGs were exposed via laminectomy using fine bone trimmers along the entire vertebral column. L3–5 DRGs were identified by following the sciatic nerve and DRGs were collected via using jewelers’ forceps to carefully grab the peripheral nerve leaving 0.5–1 cm of both peripheral nerve and dorsal root intact. Leaving the nerves intact aids in handling the tissue as it provides a surface to easily hold using forceps. This aids in transferring of DRGs during histology and mounting in the imaging chamber and prevents grabbing the cell body rich portion of the DRG, which can cause damage that readily shows up when imaging.

### 2.6. Design and materials for imaging chambers

Imaging chambers were designed and modeled in Autodesk Fusion 360. Polylactic acid (PLA), Polyethylene terephthalate glycol (PETG), Nylon, and Polypropylene chambers were printed on a heavily modified wanhao duplicator i3. PLA and PETG chambers were printed using a glass bed, nylon chambers were printed on a garolite bed, and polypropylene was printed onto (polypropylene) packing tape fixed to a glass bed (for all tested chamber materials and printing method see Table S1). Resin chambers were printed on a Formlabs, Form1 + using 3Dresyn CR UHT, or ApplyLabWork Tan resin. Chambers were designed to be leakproof and safely used with a 10x air, 20x air or 63x water immersion objective on an inverted Leica SP5 confocal microscope. This was achieved by confining the specimen to within the maximum bounds the objective could move without hitting the chamber wall. These bounds were then incorporated into the chamber design such that microscope coverslips could be securely fixed to the chamber with a suction tight seal within the chambers (Fig. S1, STL file in supplementary data). The leakproof seal was achieved by fixing the printed chambers to glass microscope slides using low viscosity cyanoacrylate glue and sanding the coverslip resting lip smooth with increasing grit wet/dry sanding paper with 150, 250, 400, 800, 1500 and 3000 fixed to a 22 mm wide flat aluminum bar. The chambers were designed to be used with 22 mm x 30 mm coverslips. Chamber outer dimensions are 27 mm wide and 35 mm long, and the inner chamber is 11 mm x 12 mm (where the tissue and submersion media are placed) with heights varying from 400 μm to 1 mm depending on the tissue to be analyzed (the minimum size chamber that the sample fit within was used). So for a 400 μm chamber 53 μL of media is needed to completely fill the chamber. Chamber materials were tested by first placing printed chambers into each of the solutions it would come in contact with and visually inspecting after 1 week for signs of deterioration (see Table S1 for more details). All chambers were tested for a leakproof seal by pressing on the center and sides of the coverslip with Pasteur pipette attached to a vacuum flask and aspirating any clearing medium until sealed (Fig. S1). Immediately following imaging, we remove the coverslip, and wash the mounting medium out of the chambers/slide, wiping it with Kim Wipes, and avoiding scratching the glass. Multiple DRGs can be imaged in one chamber, but we generally only use one at a time.

### 2.7. Tissue clearing for preserving native fluorescence

DRGs from cervical, thoracic, lumbar and sacral were dissected out and immediately cleared following the RTF (Yu et al., 2018). In brief perfusion fixed DRGs were collected and washed in PBS and then immediately placed in a solution of 30% triethanolamine (TEA), 40% formamide, (F) and 30% nanopore water (H_2_0) for 15 min at room temperature on a shake plate, then transferred to a solution of 60% TEA, 25% F, and 15%H_2_0 for 15 min at room temperature on shake plate, and finally transferred to a final solution of 70%TEA, 15% F, and 15% H_2_0 for 15–20 min (until clear). DRGs were then transferred into imaging chambers mounted on glass microscope slides.

### 2.8. Tissue clearing for immunohistology

DRG clearing and staining were performed using a slightly modified version of the iDISCO (Renier et al., 2014) and or FDISCO (Qi et al., 2019) protocols. We found that DRGs are small enough that pretreatment is not always necessary, which serves to decrease tissue processing time. The protocol followed when staining DRGs for Iba1 (Wako 019–19741, 1:1000), CD31 (BD Pharmigen 550274, 1:50), NeuN (Milipore MAB377B, 1:300), mCherry (Rockland 600–401–379, 1:1000), (Fabp7 R&D Systems, AF3166, 1:500) and DAPI (1:10,000), was as follows: All washing steps were done in 12 well plates with transfer baskets and all incubations were done in tightly sealed Eppendorf tubes or glass vials. For buffers see supplemental figures: Table S2. DRGs were washed 3 × 15 min in PTx.2, incubated in permeabilization solution for 2 h at 37 °C on shake plate, transferred to blocking solutions for 2 h (or overnight) at 37 °C on shake plate. DRGs were then incubated with primary antibody for 24–72 h at 37 °C on shake plate. If possible, DRGs can be combined into the same Eppendorf tubes to conserve reagents. If DRGs must be stained individually then a rack of PCR tubes with 200μls of solution can be used. DRGs were then washed 5 × 15 min in PTwH and incubated in secondary antibody solutions in PTwH/3% serum for 24–72 h at 37 °C on shake plate. Finally, DRGs were then washed 1x for 1–2 h in PTwH + DAPI (1:10,000), and then washed 5 × 15 min with PTwH prior to clearing. For tissue clearing we found that tetrahydrofuran (THF) often works better than methanol, but is more difficult to work with, as it can’t be stored in plastic vials. We therefore perform the THF dehydration steps in tightly sealed glass vials and dehydrate in THF/H_2_0 series as follows: 50%, 70%, 80%, 100%, 100%, then dichloromethane (DCM) 33%/THF 66%, 100% DCM and finally 100% dibenzyl ether (DBE). We observe quite a bit of shrinkage of DRGs cleared with DBE in this manner, but this actually helps in some cases by decreasing the volume of the DRG to be within the working distance of the higher magnification objectives. DRGs become completely invisible to the naked eye within seconds to minutes of being submerged in DBE so we found the best way to not lose DRGs, is to always add DAPI. This enables a weak UV light, with UV glasses, to be used to aid in visualizing the cleared DRG. DRGs can be stored long term in DBE at 4 °C or shorter periods at room temperature.

### 2.9. Imaging

Imaging was done using an inverted SP5 confocal microscope with 10x air, 20x air, and 63x water objectives. The 63x water objective used is ideal for high resolution imaging of the DRG as it’s working distance is roughly 400 μm, which can travel the entire depth of most DRGs. In order to best control for photobleaching and loss of fluorescent intensity at deeper tissue depths, we always optimized settings for the lowest laser signal intensity that effectively gave us signal and made sure that z stacks begin at the deepest tissue first and end at the most superficial.

### 2.10. Ground truth labeling and model training

We labeled images using either ilastik (Berg et al., 2019) (a commercially available interactive learning and segmentation toolkit) or in FIJI (Schindelin et al., 2012) using Labkit. In brief we made separate models for each object we wanted to segment, macrophages, DRG fiber rich region vs cell rich region, and neurons. For macrophages, an expert observer sparsely labeled signal and noise from single 2D images coming from stacks of macrophages with different stains, taken from different depths, with different intensities, and under different conditions, as a representation of any macrophage images that our model might encounter (Fig. S2). We also trained a 3D model using stacks of 30 images where 5–10 macrophages were individually labeled in every image. These models were trained for up to 2000 epochs (Fig. S2B) and resulted in highly reproducible and accurate predictions (Fig. S2C). We found that labeling single image channels and making models for individual cell types, worked far better than combining channels into RGB images and attempting to identify multiple structures at once. The same general methods were used to generate models that recognize satellite glial cells (Fig. S3). For identifying structural elements such as blood vessels, the same technique as described above was used, where ground truth labels were created by an expert observer, from images on DRGs stained for CD31. Ground truth labeling for identifying the fiber rich region (FRR) as opposed to the neuronal soma rich region (NSRR) of the DRG, was done on Iba1 stained images. Since neurons have a slightly higher autofluorescence than the fiber and a different textured appearance, one can readily distinguish between these two regions by eye with most stains (Fig. S4). To make the UNET more accurate we downsample all images to 512 × 512 pixels for training, as well as in the pipeline, and then upsample them back to their input resolution afterwards.

### 2.11. Machine Learning using YAPiC

In order to train our models, we chose to use UNET (Ronneberger et al., 2015), and train our image datasets using YAPiC (Image and Data Analysis Facility, DZNE), an open source pixel classification software. This software makes training a model and applying it to new imaging datasets very easy for anyone lacking a background in computer programing, but with basic use of bash commands. Our models were generated by training at least 2000 epochs on a P5000 GPU, RTX 2080ti or an RTX 3090. For our models we used 50 steps per epoch, the flip augmentation, local normalization, and 0.2 validation fraction on either 512 × 512 pixel images, 1024 × 1024 pixel images, or 2048 × 2048 pixel images. The output images are then converted to binary and run through the rest of the pipeline.

### 2.12. Image analysis pipeline

Our image analysis pipeline, outlined in Fig. 3, the graphical abstract and Fig. S5 is currently composed of a docker image with GPU support, which we are making freely available on our github (https://github.com/YakshLab/Yaksh-AI) to anyone who wishes to use it. The docker contains all dependencies necessary to run the analysis pipeline, which is in the form of a shell script. The shell script takes a file directory and a manifest as inputs. The manifest is a text document where each channel is named, and each UNET trained model is selected. The shell script first organizes the raw files (tif, lif, or czi currently) as described in the manifest and extracts each channel’s gray scale image. The macrophage input is then run through either a 3D UNET or 2D UNET^12^ prediction model. The resolution and scale of images should match those of the model that the image was trained on. One trick to get around this is to resample images such that the objects one wishes to identify are the same size in pixels as the objects the model was trained on. However, we would recommend generating a new model if the images were taken with different objectives or at significantly different scales. Depending on parameters set up the script will then run other images through models identified. As presented in here, the macrophage image is run through the DRG model, which predicts the fiber rich region (FRR) and the neuronal soma rich region (NSRR) of the DRG. Models were created using YAPiC (Image and Data Analysis Facility, DZNE) as described above. UNET outputs are then binarized. Binary macrophages are first filtered using a spherical erosion to help separate touching objects followed by connected component analysis using CLIJ (Haase et al., 2020) in ImageJ or clEsperanto in python. A size filter is applied where very large objects (likely multiple macrophages) are further separated using a watershed transformation, after which, they are placed back into the image map. Segmented macrophages are then classified as either NSRR, or FRR based on their location within the predicted output. The exact calculations for classification can be fined tuned by modifying the inclusion/exclusion criterion. For example, we can set an object to be included in the NSRR group if 50% or more of its pixels reside within the predicted NSRR map. Objects that aren’t classified can either be analyzed separately or removed from data analysis. A 3D map of NSRR, and FRR macrophages is then created for each image and saved for later assessment and reconstruction where each individual macrophage is displayed as a different gray value in a 32 bit image. The resulting classified macrophage regions of interest (ROIs) are then placed into the original raw images where their volume and morphological characteristics are quantified, along with the intensity characteristics of the raw image channels. Finally, all the data generated from images within the input folder is combined into one annotated spreadsheet, where each cell is labeled based on its classification and the input file name. We also analyzed each dataset using Imaris to compare our results to those generated with the commercially available software. To run this pipeline we recommend using a computer with an nvidia GPU with at least 12gb of VRAM and 64gb of RAM.

### 2.13. Comparison with existing techniques

For validation studies a macrophage expert who was not associated with DRGquant or generation of the training datasets was asked to identify and mark all macrophages in three randomly chosen 512 × 512 images stacks of 30, 50, and 100 images each. In order to compare results from the different approaches all macrophages identified, were labeled on a 2 dimensional Z projection of each image stack. Macrophages labeled using Imaris (Bitplane, Belfast UK), or using DRGquant were then compared to the label map generated by the expert. For imaris labeling a user with extensive knowledge of both Imaris and DRG macrophages manually ran each image stack using Surfaces, with surface detail of 0.5 μm, filtering voxel number set to 250 and absolute thresholding set between 12 and 25.

## 3. Results

### 3.1. Optimal materials for 3D printed imaging chambers

Through iterative design and 3D printing we were able to test imaging chambers made from many different materials (Table S1). The chambers themselves enable us to image whole mounted DRGs on an inverted confocal, without the risk of ruining expensive confocal objectives. Therefore, we first needed to find optimal materials to use for making the 3D printed chambers. We achieved the best results using a stereolithography (SLA) printer (Formlabs Form1 +) using 3Dresyns CR UHT (3Dresyns, Barcelona, Spain). These chambers were durable and easily flattened, which allows the strong seal with borosilicate coverslips necessary to contain the large volume of tissue and clearing medium. The 3Dresyns CR UHT chambers failed to degrade after over 100 uses and never fell off the slide when glued with low viscosity cyanoacrylate (Fig. S1). For fused deposition modeling (FDM) printers we initially thought that nylon would be the best material, as it is the most chemically resistant, however, nylon printed chambers did not adhere well to glass microscope slides. Instead, we found that polypropylene printed chambers were superior when using an FDM printer. Polypropylene chambers lasted 30–80 uses before falling off, so we recommend ensuring that they are securely attached to the slide before mounting tissue (Table S1).

### 3.2. DRG histology of expressed fluorescent protein vs. immunostaining

**Fig. 1.**
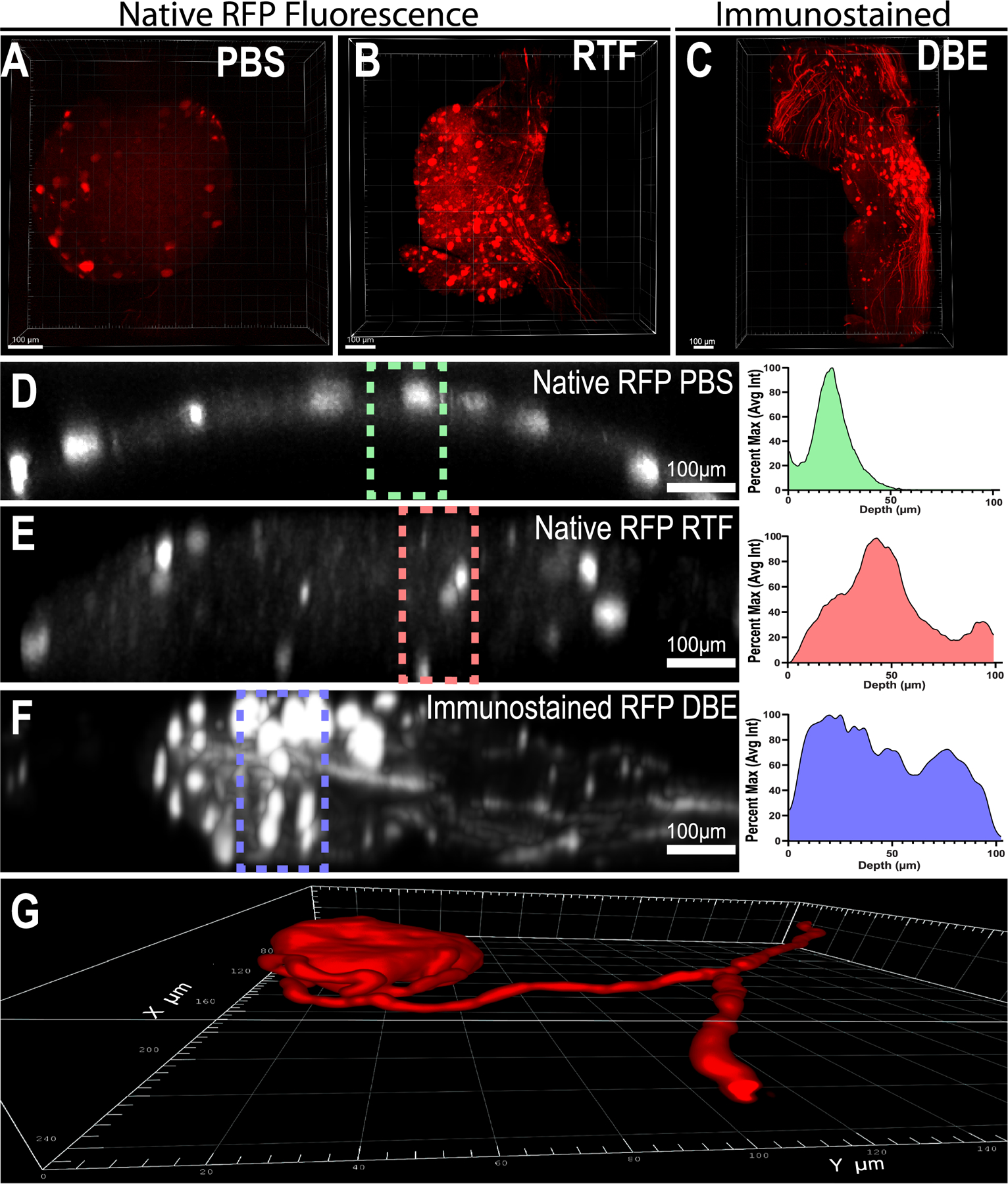
*Virally Transfected DRG Neurons. (*A-C) Representative projections of AAV9-mCherry transfected DRG neurons. (A) Native fluorescence of RFP without tissue clearing in PBS. (B) Native Fluorescence of RFP after tissue clearing with RTF. (C) Immunostained DRG cleared with DBE. Scale bar 100 μm. (D-F) Representative orthogonal z projections showing the depth of imaging, with adjacent histogram showing average intensity within box.(D) Native RFP without clearing. (E) Native RFP with RTF clearing (F) Immunostained RFP cleared with modified DISCO/DBE. Scale bar 100 μm. Histograms in D-F show average intensity within bounding area displayed as percent of max average intensity as a function of imaging depth. (G) Reconstruction of native fluorescence from virally transfected neuron illustrating the glomerular structure of the extending axon and tracing the axon to the point of bifurcation and beyond.

**Fig. 2.**
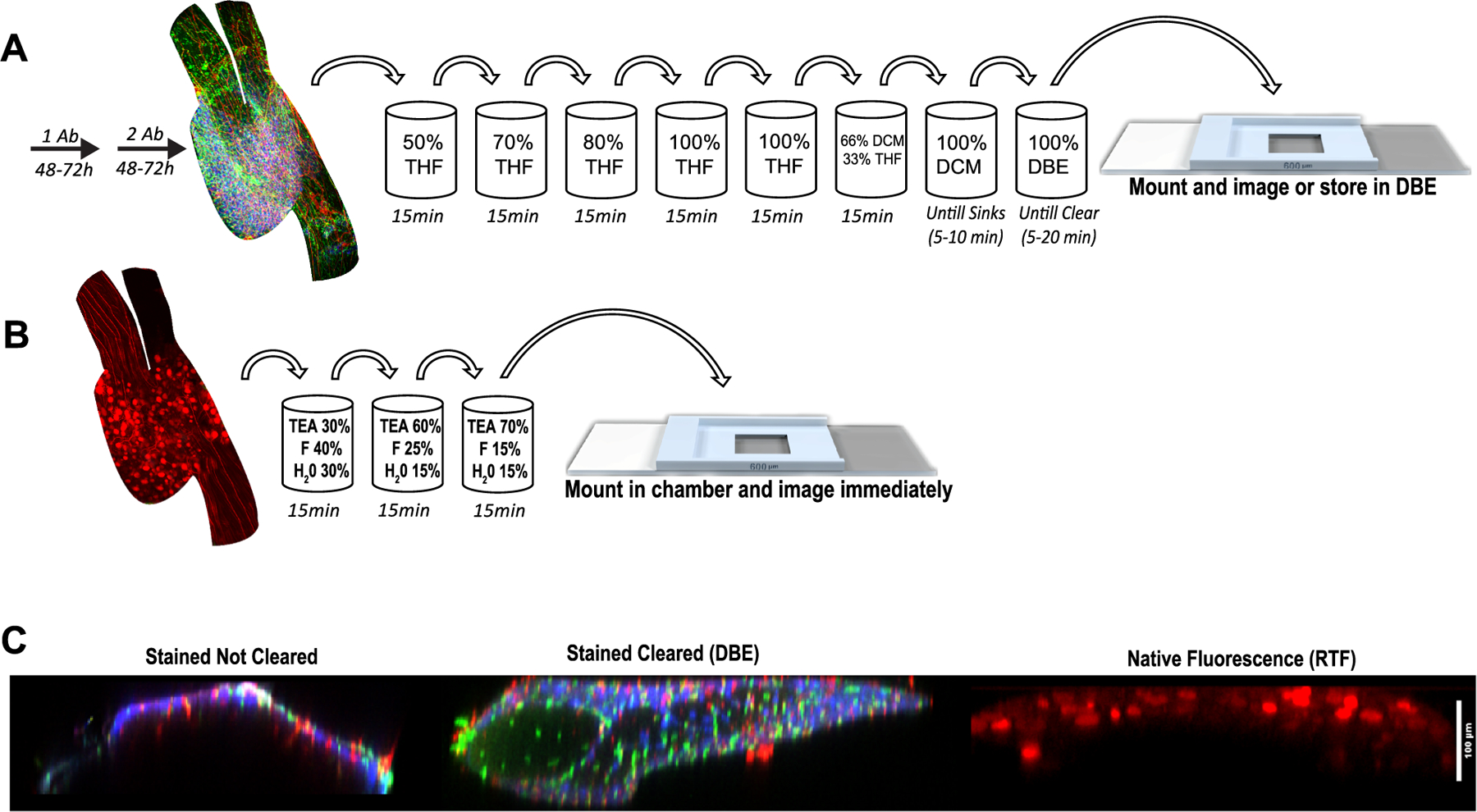
*DRG Clearing Protocols.* (A) Process for immunohistochemistry showing our abbreviated version of iDISCO. (B) Process for imaging endogenous fluorescence of virally transfected DRG neurons expressing RFP. (C) Comparisons between stained but not cleared DRG, a stained DRG cleared with DBE, and the native RFP fluorescence of a DRG cleared with RTF. DRGs are stained with Iba1 (macrophages, red), NeuN (neurons, blue), and CD31 (endothelium, green). All images were taken at 10x magnification and displayed is the XY axis with scale bar 100 μm.

We then set out to optimize protocols for imaging endogenous fluorescence, as well as immunostained DRGs. When analyzing fluorescence of DRG neurons that were virally transduced to express RFP, we found that many of the available clearing techniques quenched the fluorophore. We then found that the RTF (Yu et al., 2018) method (rapid clearing method based on Triethanolamine and Formamide) (Fig. 1; Table S3) preserved native fluorescence of RFP well, while being the fastest of the methods we tested. Using RTF on whole DRGs to assess native fluorescence of RFP labelled neurons we were able to get a good depth of signal (> 100 μm) using confocal microscopy. Additionally using this method, we could have whole DRGs cleared and imaged at10x magnification within 1 hr of collection, resulting in high quality images (Fig. 1B and C, Movie S1 and S2). Virally labeled DRG neurons could be imaged at a high resolution for accurate reconstructions of DRGs and tracing of individual afferent axons, even to their points of bifurcation and beyond (Fig. 1D, Movies S1–S3). While RTF doesn’t cause tissue shrinkage like many of them methods that involve dehydration, tissue clarity was not as good as other methods we tried, and immuno-stains were often quenched when left in RTF solutions. We modified a commonly used iDISCO protocol (Renier et al., 2014) as described in detail in the methods above (Fig. 2A and B) which yielded better quality, greater clarity, and increased signal to noise ratio over RTF, especially when staining the large L4 mouse DRGs (Fig. 2C; Table S3). Therefore, for all immunohistology we followed the protocol outlined in Fig. 2A.

Supplementary material related to this article can be found online at doi:10.1016/j.jneumeth.2022.109497.

### 3.3. Pipeline for 3D image analysis of DRG macrophages

As noted, we have a great interest in macrophages and nociceptive processing but found it difficult to accurately quantify their activation using traditional methods. Even after developing our whole mount techniques we found it difficult to analyze these large 3D datasets using freely available software such as ImageJ (Schneider et al., 2012). The limiting factor lies in the difficulty of normalizing the signal and noise intensities over an entire stack of images and finding a way of thresholding so that individual cells are isolated and represented. We were, however, able to manually segment and quantify macrophages when using software such as Imaris (Bitplane, Belfast UK). However, most available software/techniques require manual setting of thresholds for each image stack, which introduces a significant potential for inadvertent bias. Therefore, we turned to machine learning to as a strategy to develop an agnostic algorithm to effectively isolate signal from noise in image stacks.

**Fig. 3.**
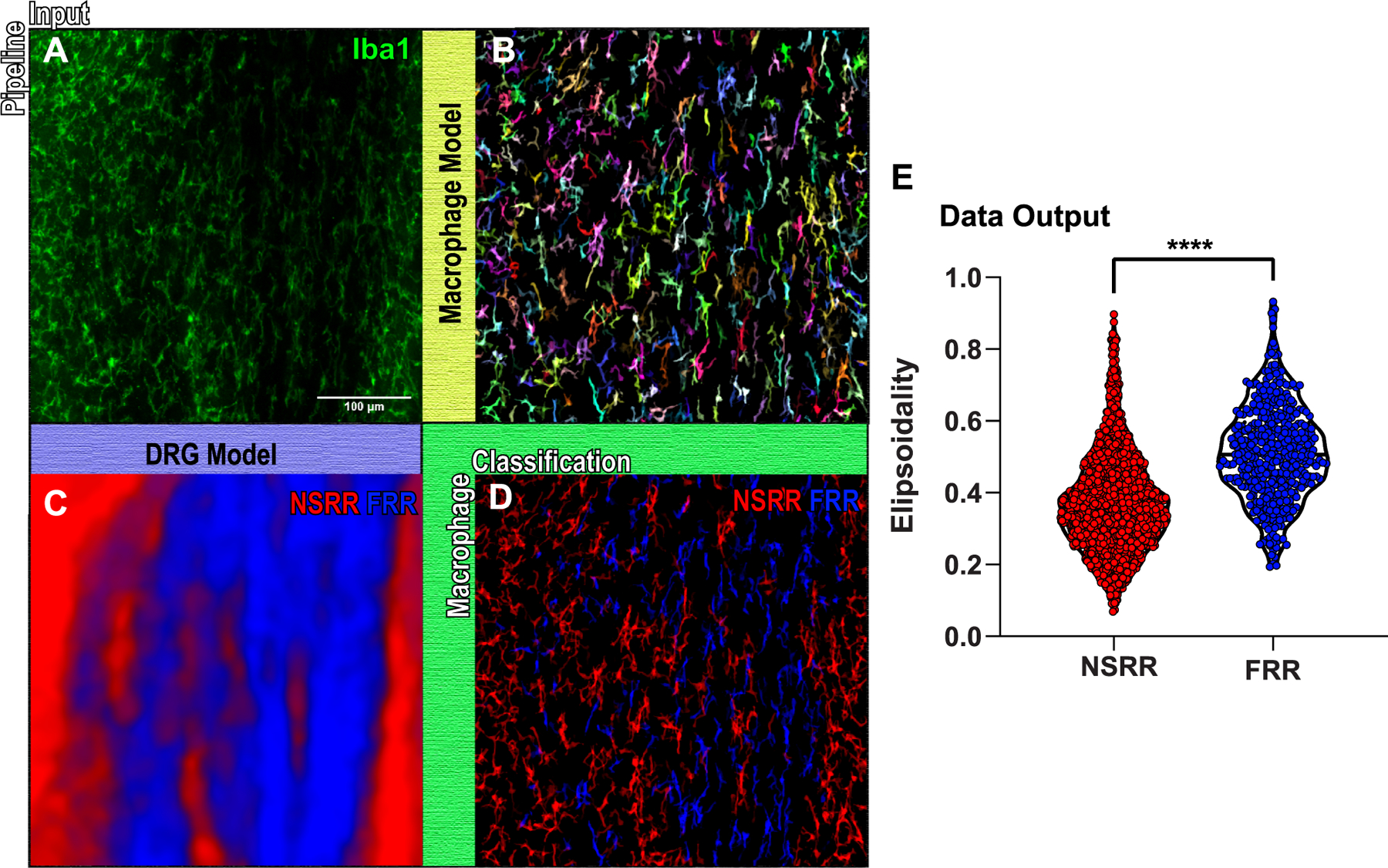
Image analysis pipeline example: segmentation and identification of DRG macrophages. A) Shows a z projection of an Iba1 stained input image from a representative, difficult to manually threshold, wholemount DRG. The full raw Iba1 stack is then run through both the trained macrophage model (B) as well as the NSRR/FRR model (C). B.) Z projection of identified macrophages where each cell is randomly colored. C) Predicted z projection map of the Fiber Rich Region (FRR, blue) and the Neuronal Soma Rich Region (NSRR,red). D) The classification of individual macrophages based on their location within the DRG models’ output. Scale bar is 100 μm. E) Example of data output is given showing that FRR macrophages are more ellipse shaped as compared to NSRR macrophages within the DRG (unpaired Student *t* test;****P < 0.0001).

*DRGquant,* as shown here, first runs a z stack of raw Iba1 stained macrophages through a model trained to identify macrophages (Fig. 3A and B, Movie S4), then runs the same raw image through a model trained to identify the fiber rich region of the DRG (FRR) and the neuronal soma rich region (NSRR) of the DRG (Fig. 3C). DRGquant can then classify individual macrophages as either NSRR macrophages or FRR macrophages (Fig. 3D). Our interest in this classification were two-fold. First, we wanted a better way to normalize cell number to tissue volume. This is especially important in sectioned DRGs, where large regions of the section may be taken up by the FRR, but is also incredibly important in whole mount DRGs. Secondly, we were interested in this classification from observation that macrophages in the fiber rich region (FRR) of the DRG had a very different morphology (long and linear) compared to the more heterogeneously shaped DRG macrophages in the NSRR which we were crescent shaped and linear, as well as more complicated morphology (Fig. 3E). The pipeline then populates data tables for each cell as well as a 32-bit label map, where each cell is given a unique gray scale value. We can then set a look up table (LUT), for example “glasbey on dark” in ImageJ, which randomly assigns colors to each gray value that can be used post hoc to visualize the results as either 2D z projections (Fig. 3B) or 3D reconstructions (Movie S2). This serves to assess the quality of macrophage segmentation and enables visual identification of whether multiple cells became clumped together, or if cells were split apart.

Supplementary material related to this article can be found online at doi:10.1016/j.jneumeth.2022.109497.

### 3.4. Validation of macrophage segmentation

**Fig. 4.**
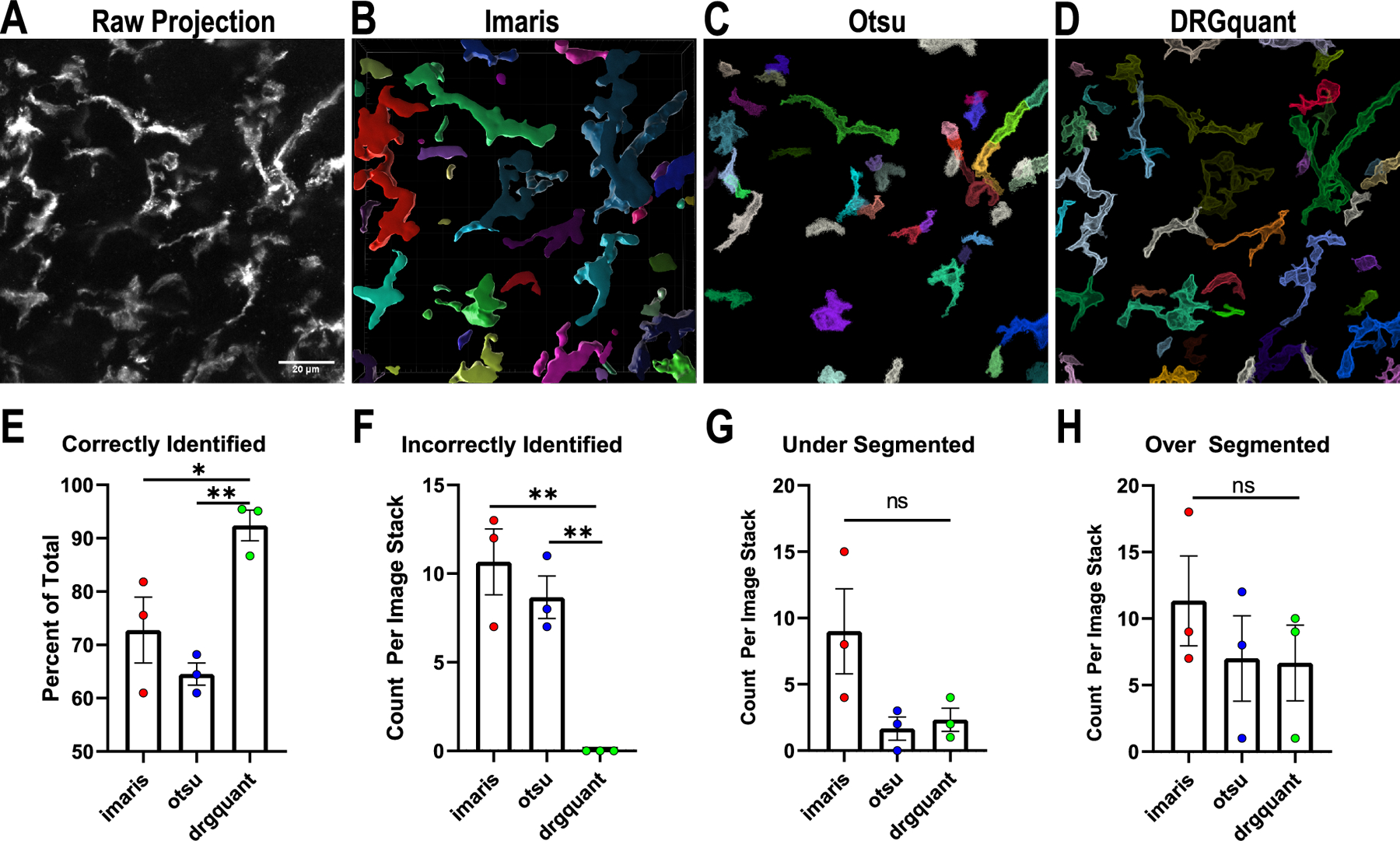
*DRGquant Validation.* A) Shows a z projection of an Iba1 stained image stack, where each individual macrophage was identified by an exert observer. Scale bar is 20 μm. Reconstructions of macrophages from A where each object is depicted with a different color when analyzed with B) Imaris C) Otsu thresholding replacing UNET Model in the DRGquant pipeline and D) DRGquant. E) Correctly identified macrophages shown as percent of total macrophages identified by an expert. F) Incorreclty identified macrophages characterized as objects detected that were not determined to be macrophages or objects identified as macrophages that were not detected and displayed as the count per image stack. G) Under segmented macrophages were characterized as multiple macrophages identified as one object and displayed as count per image stack. H) Over segmented macrophages were multiple objects detected that were identified as a single macrophage by an expert and displayed as count per image stack (one way ANOVA with Tukey correction for multiple comparisons; *P < 0.05, ** P < 0.01, ns=not significant).

In order to assess the quality of DRGquant’s macrophage segmentation we chose 3 512 × 512 pixel substacks consisting of 30, 50 and 100 images stained with Iba1 to identify macrophages. The image stack with 50 images is displayed as a 2D z projection (Fig. 4A). An independent macrophage expert was asked to manually identify all individual macrophages within each image stack of the dataset. The dataset was then analyzed using Imaris (Fig. 4B) or using DRGquants pipeline (Fig. 4C–D). As DRGquant uses UNET to aid in thresholding we also tested DRGquant’s pipeline with a simple Otsu thresholding replacing the UNET trained macrophage model prediction (Fig. 4C). DRGquant significantly outperformed Imaris and Otsu thresholding in correctly identifying DRG macrophages (Fig. 4E). Further, in all three image stacks DRGquant failed to incorrectly identify a single macrophage (Fig. 4F). DRGquant performed equally well to Imaris and Otsu thresholding with regards to Under and Over segmentation of macrophages (Fig. 4G–H). The time it took to run the full dataset was 3.87 s for DRGquant with Otsu thresholding, 33 s for the full DRGquant pipeline, and 50 min to analyze using Imaris.

### 3.5. DRG Macrophage activation following intrathecal lipopolysaccharide

**Fig. 5.**
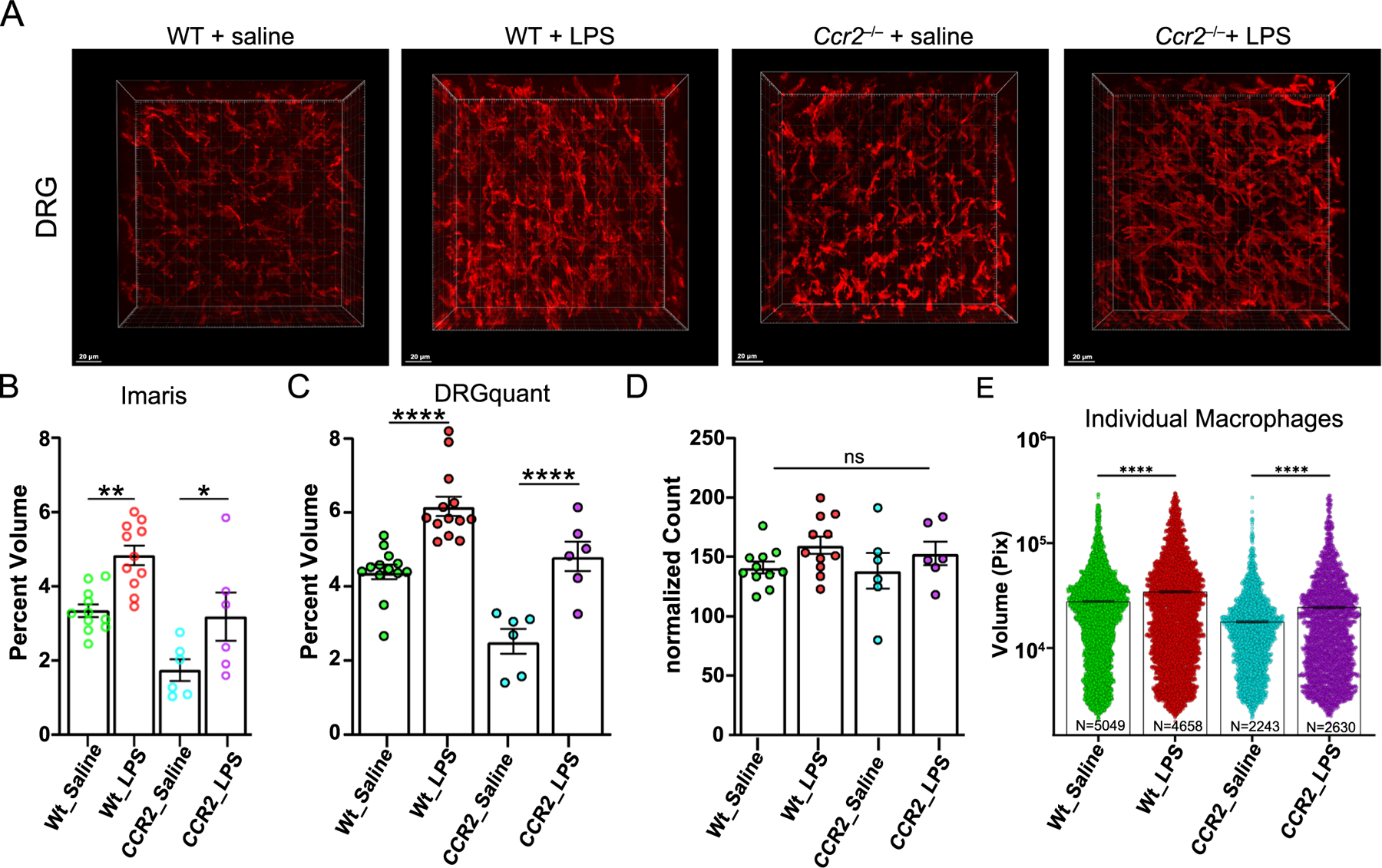
LPS induced macrophage activation in our pipeline comparison. (A) Representative images of macrophages 24 h after intrathecal saline or LPS in wild type C57BL/6 mice and *Ccr2*^−/−^ mice. Scale bar 20 μm. (B) LPS induces an increase in the volume over macrophages over total volume of the sample in both wild type and *Ccr2*^−/−^ mice, Analysis performed with Imaris. (C) Automated analysis with our pipeline shows consistent results with Imaris. (D) No difference is observed between PV and NPV macrophages in macrophage numbers. (E) Using DRGquant we can see that individual macrophages increase in size following IT-LPS in both wild type and CCR2KO mice (one way ANOVA with Tukey correction for multiple comparisons; *P < 0.05, ** P < 0.01, ***P < 0.001, **** P < 0.0001, ns = not significant, number of mice: Wt_saline=6, Wt_LPS=6, CCR2^−/^_saline=3,CCR2^−/−^_LPS=3, 2 DRGs per mouse).

To validate our methods and pipeline further, we employed intrathecal (IT) lipopolysaccharide (LPS), which induces a profound allodynic pain state accompanied by an increased DRG macrophage signal (Raoof et al., 2020; Zhang et al., 2016). Using our whole mount imaging, we could readily identify a qualitative difference between wild type C57BL/6 mice with IT LPS as compared to those with IT saline (Fig. 5A–C). In order to determine if the observed difference was due to infiltration of macrophages into the DRG we injected *Ccr2*^−/−^ mice with IT LPS. Lacking the C-C chemokine receptor type 2 (CCR2) renders monocytes and macrophages incapable of being recruited during immune responses, thus, indicating whether an increase in macrophages was observed due to infiltration of circulating macrophages, local proliferation, or macrophage expansion (Kurihara et al., 1997). In analyzing 63x image stacks with the *DRGquan*t pipeline, we found that the percent volume of macrophages in the DRG increases following IT LPS in both wild type and Ccr2^−/−^ mice (Fig. 5B and C), highlighting an increase of volume of resident macrophages. We validated *DRGquant* by reanalyzing our images with Imaris, a software solution for interactive image analysis, and got similar overall results (Fig. 5B and C). Further, using *DRGquant* we could determine how many individual macrophages were actually present in each sample. This approach demonstrated that, in contrast to a prevalent literature(Lindborg et al., 2018; Lu and Richardson, 1993; Yu et al., 2020) there was in fact *no increase* in the number of DRG macrophages following IT LPS (Fig. 5D), a finding consistent with the results we observed with the Ccr2^−/−^ mice (Fig. 5D). Interestingly, we could also analyze the size of individual Iba1 positive macrophages and observed a change in percent volume of macrophages in the DRG (Fig. 5E).

### 3.6. DRG macrophage activation following cisplatin induced peripheral neuropathy

**Fig. 6.**
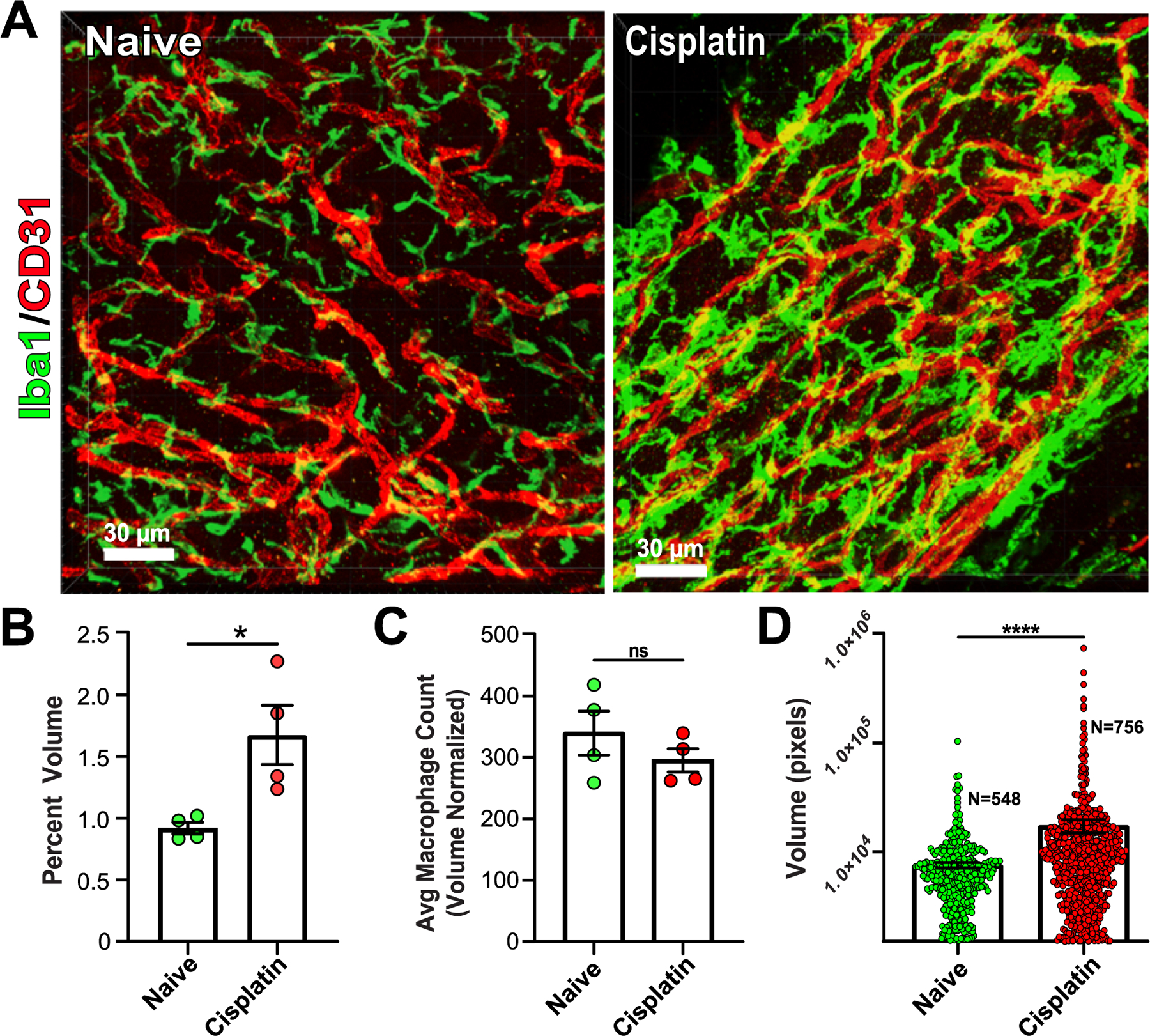
Macrophage activation following cisplatin induced CIPN. (A) Representative 3D reconstruction images of mouse DRGs collected from naive (left) and 10 days after cisplatin injection (right) scale = 30 μm. (B) Mean percent volume of macrophages increases following cisplatin. * P < 0.05. (C) Average macrophage count normalized by volume does not change following cisplatin. (D) The volume of individual macrophages increase following cisplatin (two tailed Mann-Whitney test; ns = not significant, ****P < 0.0001, number of mice: Naïve=4 Cisplatin=4).

Following our observation that macrophage size increased following ITLPS but not macrophage number, we decided to use our methods and DRGquant on a model with well-established macrophage infiltration/increase in number, chemotherapy induced peripheral neuropathy (CIPN). DRGs collected from mice 10 days after cisplatin displayed a robust increase in Iba1 staining as compared to mice treated with saline (Fig. 6A). Just as with ITLPS we observe a large increase in the percent volume of the DRG (Fig. 6B). Interestingly, in contrast to nearly all published literature, we did not observe an increase in the number of DRG macrophages following CIPN (Fig. 6C). Finally, we observed a very large increase in the volume of individual macrophages from mice that were given cisplatin (Fig. 6D).

### 3.7. DRG macrophage uptake of fluorophore labelled dextran

**Fig. 7.**
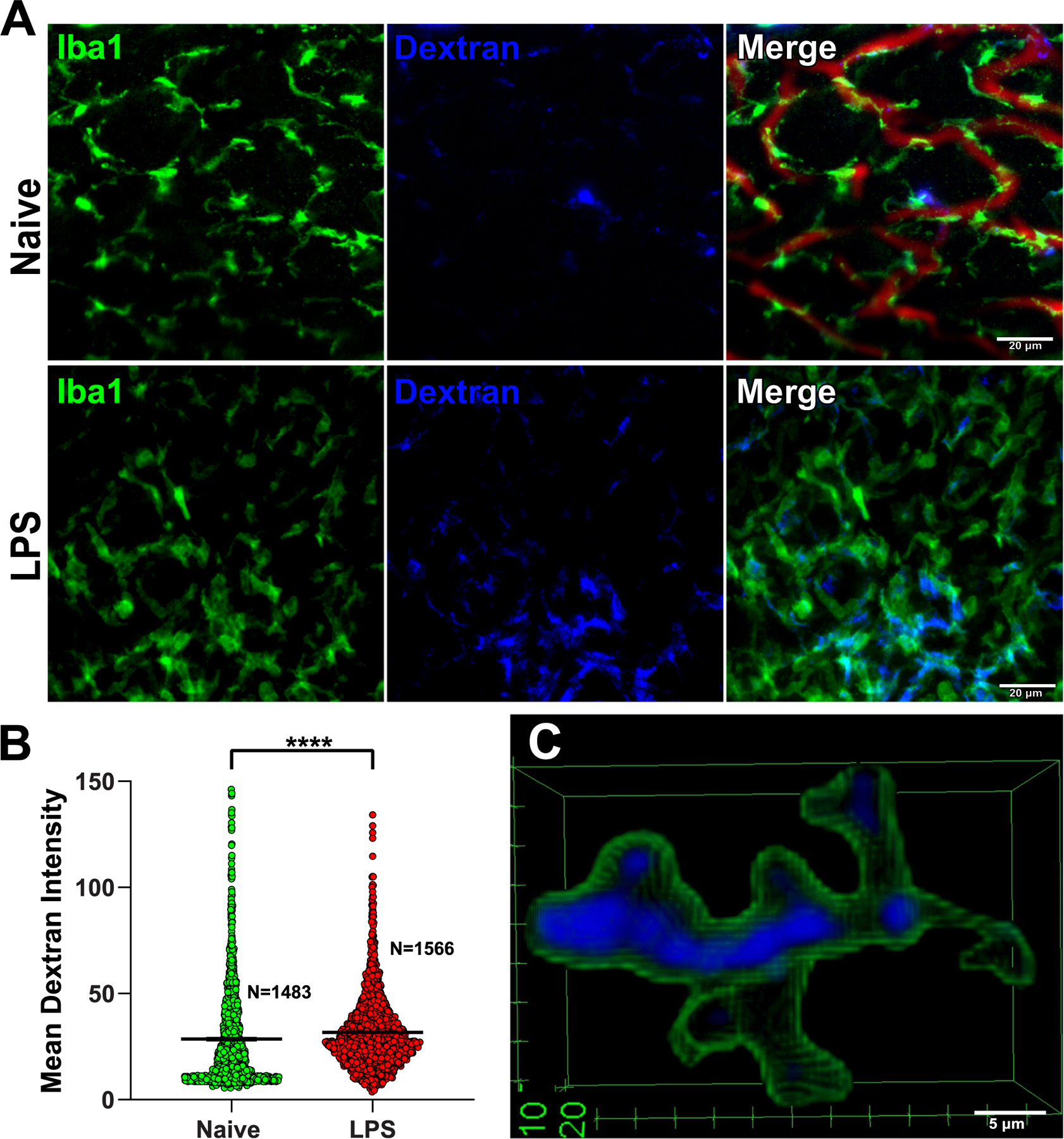
Uptake of fluorophore labeled dextran. (A) Representative sum z stack images of naïve (top) and 24 h following IT LPS injected (bottom) whole mount DRGs stained with Iba1 for macrophages, dextran, and CD31 for blood vessels. Scale bar 20 μm (B) Mean intensity of dextran within individual macrophages in the DRGs of naïve or IT LPS injected mice (one way ANOVA with Tukey post hoc test, means and individual macrophages are shown; **** P < 0.0001). (C) 3D reconstruction of single macrophage with 3D ROI of *DRGquant* output outline in green and dextran in blue, showing subcellular spatial resolution. Scale bar is 5 μm.

To further display the power of our technique we used fluorophore labeled 70 kDa dextran administered intravenously as an indicator of the DRG macrophages accessibility to circulating agents. We found that macrophage uptake of fluorophore labeled dextran is detectable and quantifiable using the DRGquant pipeline (Fig. 7A–C). The uptake is significantly greater in macrophages from mice injected with IT LPS (Fig. 7B). In addition, 3D reconstruction of single macrophage with 3D ROI of *DRGquant* demonstrates subcellular spatial resolution (Fig. 7C, also see Movie S5 and Movie S6).

Supplementary material related to this article can be found online at doi:10.1016/j.jneumeth.2022.109497.

## 4. Discussion

The tissue preparation protocols and analytic pipeline we describe here enables rapid preparation of DRG tissues to develop optical image stacks that can be reconstructed for 3D presentation (see supplemental movies).

The quantitative analysis presented here was possible due to the development of a modular artificial intelligence-based pipeline for 3D analysis of the DRG. Our results show the power of using machine learning to separate signal from noise and identify individual cells, as well as isolate individual structures or areas of interest within the DRG for a more granular and fine-tuned analysis (for further examples see movie S7 and movie S8 as sample where blood vessels are isolated as a chosen DRG structure). Further, our approaches and tools, using the open access platform as described can be implemented by anyone and additional AI models can be trained using any annotated image stack.

Supplementary material related to this article can be found online at doi:10.1016/j.jneumeth.2022.109497.

In the present work, we performed proof of principle experiments that show the utility and functionality of this pipeline focusing on macrophages and their location within the DRG. Our initial interest in the disposition of this cell type arose through observation that FRR macrophages had a distinctly different morphology, and were often longer and straighter than NSRR macrophages which usually appear to have more of a crescent shape (likely due to surrounding a neuronal soma). Future experiments will be able to compare FRR and NSRR macrophages under various circumstances to identify whether these are indeed two different sub-populations of macrophages occupying the same niche environment.

Often infiltration of macrophages has been described in the literature following injury (Hu and McLachlan, 2003; Simeoli et al., 2017), as well as following paclitaxel (Zhang et al., 2016), vincristine (Peters et al., 2007) and cisplatin (Laumet et al., 2019) induced peripheral neuropathy. Treatment of rats with minocycline (suppressing macrophage/microglia activation) has been shown to alleviate hyperalgesia following oxaliplatin induced peripheral neuropathy (Boyette--Davis and Dougherty, 2011). Many of these experiments have used traditional techniques of quantifying macrophages (pixel density, counting of thin section profiles) and often conclude that increased infiltration, and or increased numbers of macrophages are present. Thus unexpectedly, given the extant literature, in none of the experiments and quantifications performed here, did we observe significant differences in the number of macrophages in DRGs following any treatments. This result is consistent with our results from LPS injection with the Ccr2^*−/−*^ mice, which should have no infiltration of monocytes or macrophages into the DRG. This work suggests that one potential pitfall with traditional histology approaches is the amorphous organization and orientation of macrophages, which leads thin section histology to observe more immunoreactive macrophage-like profiles, when in fact there may simply be larger cells with more processes that pass in and out of the plane of section. These results are important as it raises the likelihood that depletion of peripheral circulating monocytes may have little influence on the role played by the DRG-resident macrophage.

Two points are made in conclusion. First, the results obtained using DRGquant were superior to those obtained using currently available software, validating our analysis strategy. Software designed for 3D image analysis such as Imaris, while useful, can be both expensive, and requires extensive user input in setting thresholds for each individual image stacks. This leaves the user with the possibility of introducing bias, even when blinded. For instance, it is often easy to distinguish between LPS and saline treated DRG macrophages qualitatively simply by viewing the resulting images (see Figs. 6 and 7), which is something a user must do when manually setting thresholds. The use of machine learning by the *DRGquant* pipeline eliminates potential user bias from the analysis, and yet obtains comparable results in terms of changes in percent volume of macrophages. Secondly, while the experiments presented here focused on macrophages, one can readily change analytic parameters and cell types due to the modularity of this approach (See Fig. S4 for satellite glial cells and movie S8). For instance, we could explore if macrophages in contact with nociceptive neurons expressing the protein calcitonin gene related peptide (CGRP) are changing in response to pain-states, one would simply stain CGRP positive neurons and instead of isolating macrophages in the NSRR and FRR of the DRG, the pipeline would classify macrophages in contact with CGRP positive neurons and structures. We have also imagined this pipeline will be used to combine multiple stains of macrophages to get flow cytometry quality data, but retaining morphological characteristics and cell quantity as well. This is, in essence, what we show in the final experiments with dextran, where we can observe uptake within macrophages. If one took this to the logical extreme, we could stain 6 or even potentially up to 10 markers at a time and use one strong, non-overlapping signal to identify your cell type of interest (e.g. Iba1 here). In summary we present a robust protocol for whole mount preparation and staining of cleared DRG and a reliable AI tool for analysis.

## Supplementary Material

Supplemental Figures

Movie 1

Movie 2

Movie 3

Movie 4

Movie 5

Table S1

Table S2

Table S3

Figure S1

Figure S2

Figure S3

Movie 6

Movie 7

Movie 8

## Abbreviations:

CIPN: chemotherapy induced peripheral neuropathy
CIS: cisplatin
FDM: fused deposition modeling
FRR: fiber rich region
LPS: lipopolysaccharide
LUT: look up table
NSRR: neuronal soma rich region
PETG: polyethylene terephthalate glycol
PLA: polylactic acid
ROI: region of interest
RTF: rapid clearing method based on triethanolamine and formamide
SAL: saline
SLA: stereolithography
TEA: triethanolamine
THF: tetrahydrofuran
