# Supplementary material for: DRGquant: A new modular AI-based pipeline for 3D analysis of the DRG
Source: J Neurosci Methods. Author manuscript; Available in PMC 2023 Nov 14. (PMC10644910; doi:10.1016/j.jneumeth.2022.109497)
Supplement: Supplemental Figures [file NIHMS1792468-supplement-Supplemental_Figures.docx]

Supplemental Figures/Tables


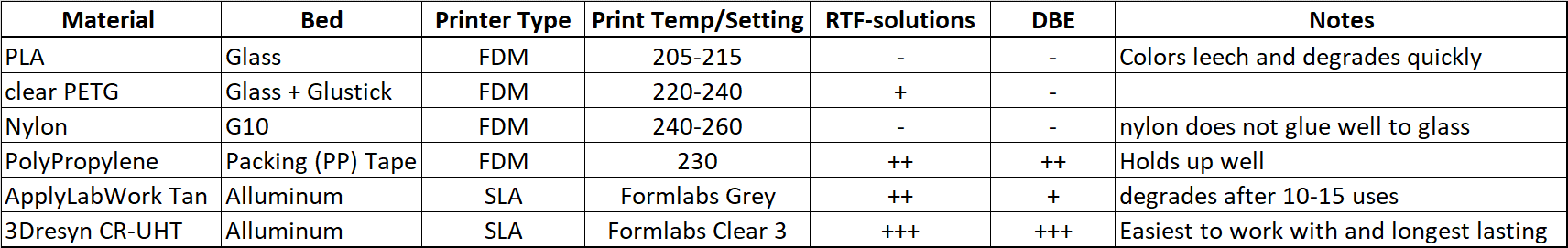


Table S1 : Chamber materials


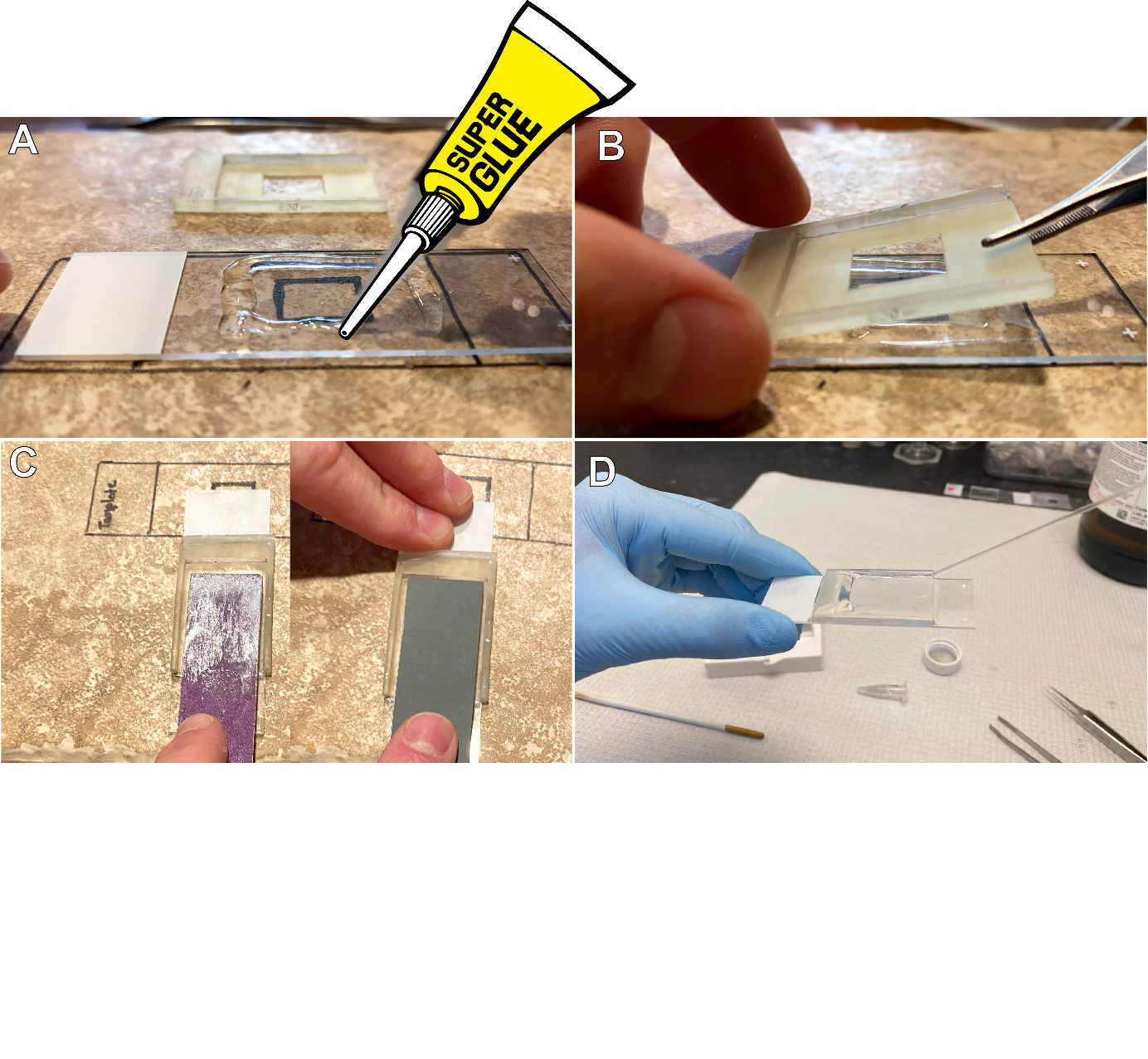


Figure S1 Making DRG chambers: chambers are printed in a resin SLA printer. (A) Glue is placed on a microscope slide (B) The chamber is then attached (C) Chambers are flattened and smoothed to make sure they will form a perfect seal with sandpaper grits from 200-3000. (D) Tissues are mounted in their respective clearing medium and then any additional medium is removed via aspiration with a fire polished Pasteur pipette affixed to a vacuum flask.

| **Histology Solutions** | **PBS-Az** | 100mL 10x PBS  0.2g Sodium Azide (NaN_3_) 900mL Nanopure H20 |
| --- | --- | --- |
|  | **PTx.2** | 2 mL TritonX-100  998 mL PBS-Az |
|  | **PTwH** | 2 mL Tween-20  1 mL Heparin (10mg/ml) |
|  | **Permeabilization Solution** | 23g Glycine 200 mL DMSO 800 mL Ptx.2 |
|  | **Blocking Solution** | 3 mL Serum 5 mL DMSO 42 mL PTx.2 |

Table S2: Histology Solutions

| **RTF (Native Fluorescence)  Clearing Solutions** | **RTF1** | Triethanolamine-30% Formamide-40%  Water-30% |
| --- | --- | --- |
|  | **RTF2** | Triethanolamine-60% Formamide-25%  Water-15% |
|  | **RTF3** | Triethanolamine-70%  Formamide-15%  Water-15% |

Table S3 : Solutions for clearing DRGs to preserve native fluorescence


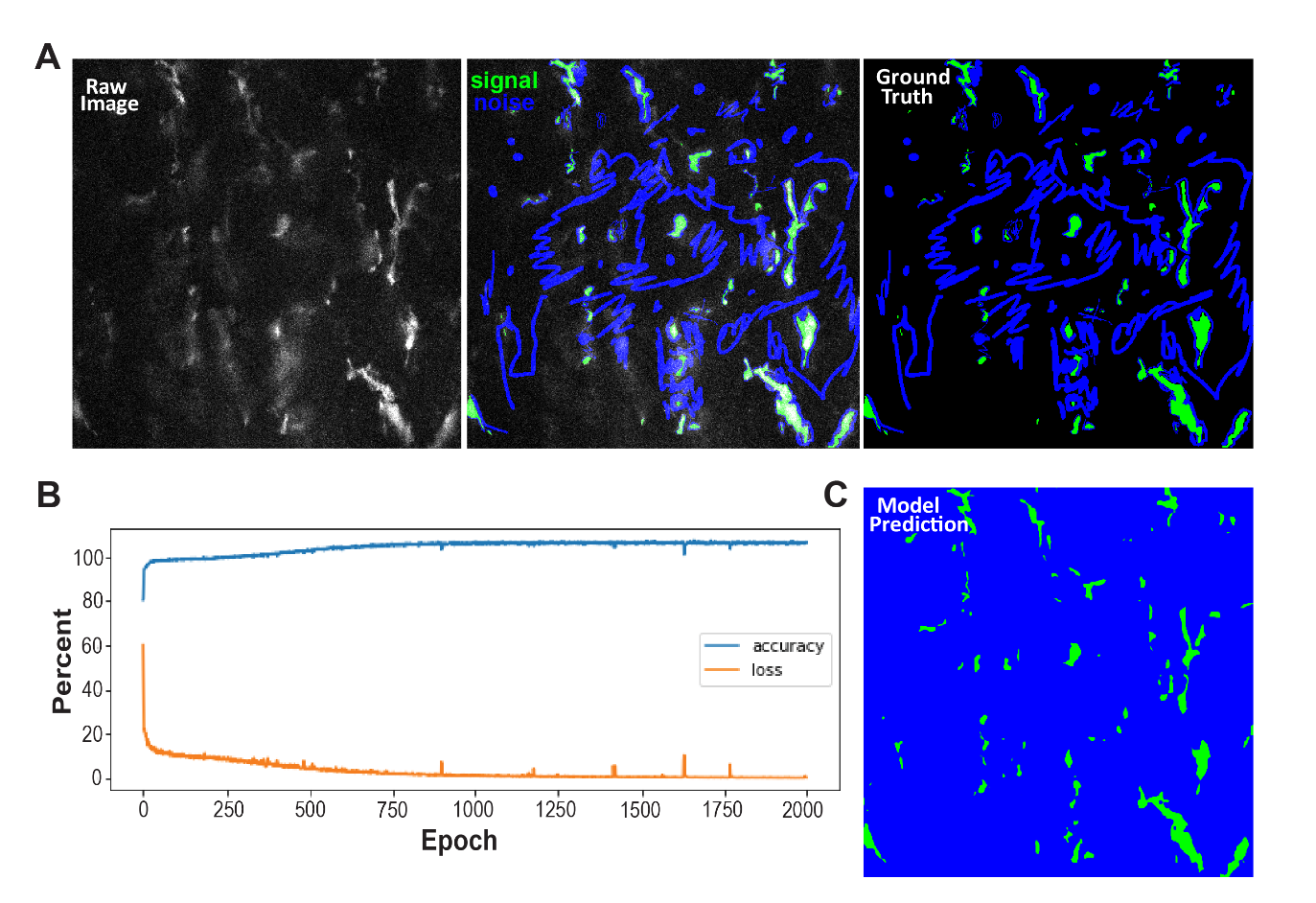


Figure S1 Macrophage training A.) Sample of raw grayscale image of Iba1 stained macrophages in the DRG. Signal (green) and background is manually drawn by an expert. True signal vs noise then becomes a set of ground truth images for each raw image. B.) Training of this model was done for 2000 epochs showing accuracy increasing as loss decreases. C.) The results of running the raw image through the trained model where blue is background and green is macrophage signal.


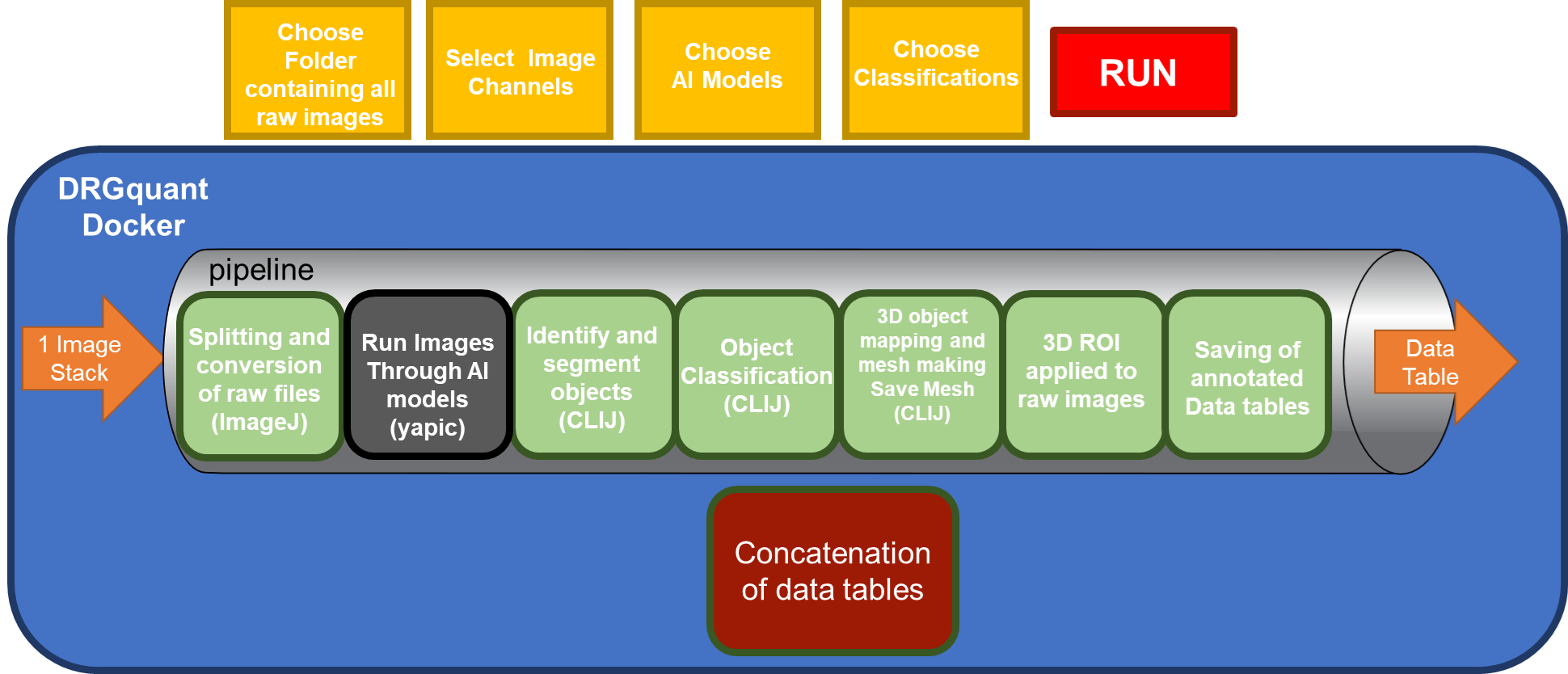


Figure S3 Flow chart of image analysis pipeline: Flow chart displays the inputs, outputs, and steps involved in the DRGquant pipeline

Stl file for chambers

Movie 1 = bifurcating neuron movie

Movie 2 = America neurons?

Movie 3 = segmented macrophages

Movie 4 = macrophages with Dextran

Movie 5 = individual macrophage with Dextran
