# Supplementary material for: DRGquant: A new modular AI-based pipeline for 3D analysis of the DRG
Source: J Neurosci Methods. Author manuscript; Available in PMC 2023 Nov 14. (PMC10644910; doi:10.1016/j.jneumeth.2022.109497)
Supplement: Table S1 [file NIHMS1792468-supplement-Table_S1.pdf]

Table S1 : Chamber materials

Material	Bed	Printer Type	Print Temp/Setting	RTF-solutions	DBE	Notes
PLA	Glass	FDM	205-215	-	-	Colors leech and degrades quickly
clear PETG	Glass + Glustick	FDM	220-240	+	-	
Nylon	G10	FDM	240-260	-	-	nylon does not glue well to glass
PolyPropylene	Packing (PP) Tape	FDM	230	++	++	Holds up well
ApplyLabWork Tan	Alluminum	SLA	Formlabs Grey	++	+	degrades after 10-15 uses
3Dresyn CR-UHT	Alluminum	SLA	Formlabs Clear 3	+++	+++	Easiest to work with and longest lasting
