# Supplementary material for: DRGquant: A new modular AI-based pipeline for 3D analysis of the DRG
Source: J Neurosci Methods. Author manuscript; Available in PMC 2023 Nov 14. (PMC10644910; doi:10.1016/j.jneumeth.2022.109497)
Supplement: Table S2 [file NIHMS1792468-supplement-Table_S2.pdf]

Histology Solutions		
	**PBS-Az**	100mL 10x PBS<br>0.2g Sodium Azide (NaN_3)<br>900mL Nanopure H20
	**PTx.2**	2 mL TritonX-100<br>998 mL PBS-Az
	**PTwH**	2 mL Tween-20<br>1 mL Heparin (10mg/ml)
	**Permeabilization Solution**	23g Glycine<br>200 mL DMSO<br>800 mL Ptx.2
	**Blocking Solution**	3 mL Serum<br>5 mL DMSO<br>42 mL PTx.2
