# Supplementary material for: DRGquant: A new modular AI-based pipeline for 3D analysis of the DRG
Source: J Neurosci Methods. Author manuscript; Available in PMC 2023 Nov 14. (PMC10644910; doi:10.1016/j.jneumeth.2022.109497)
Supplement: Table S3 [file NIHMS1792468-supplement-Table_S3.pdf]

**RTF (Native Fluorescence) Clearing Solutions**	**RTF1**	Triethanolamine-30%<br>Formamide-40%<br>Water-30%
	**RTF2**	Triethanolamine-60%<br>Formamide-25%<br>Water-15%
	**RTF3**	Triethanolamine-70%<br>Formamide-15%<br>Water-15%
