# Supplementary figures and images for: DRGquant: A new modular AI-based pipeline for 3D analysis of the DRG
Source: J Neurosci Methods. Author manuscript; Available in PMC 2023 Nov 14. (PMC10644910; doi:10.1016/j.jneumeth.2022.109497)

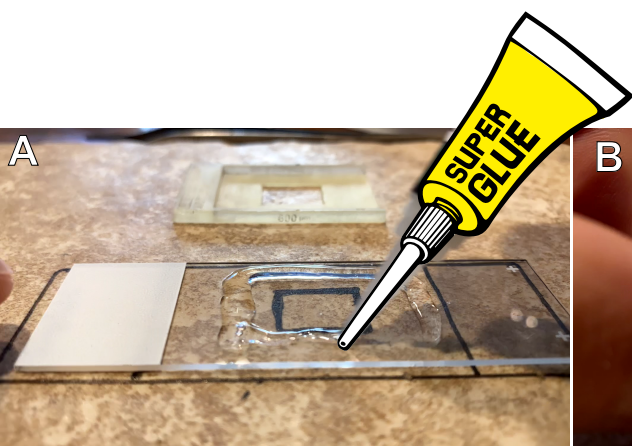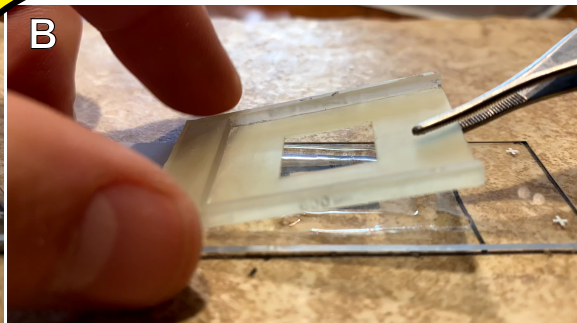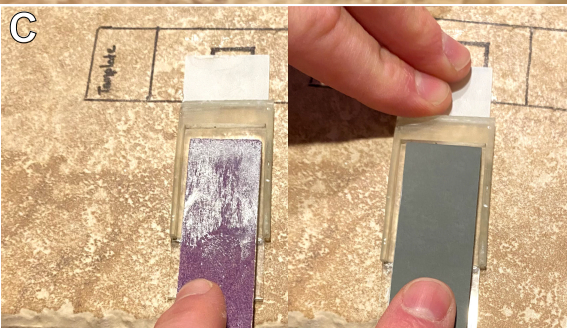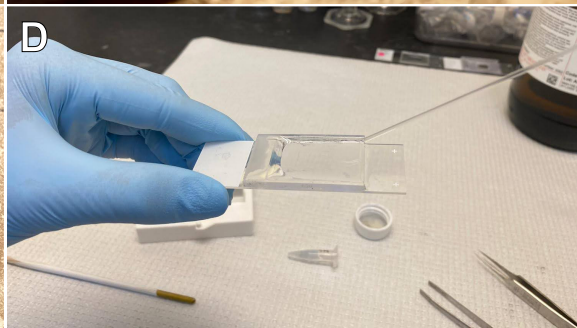

Supplement: Figure S1 [file NIHMS1792468-supplement-Figure_S1.pdf]

**A**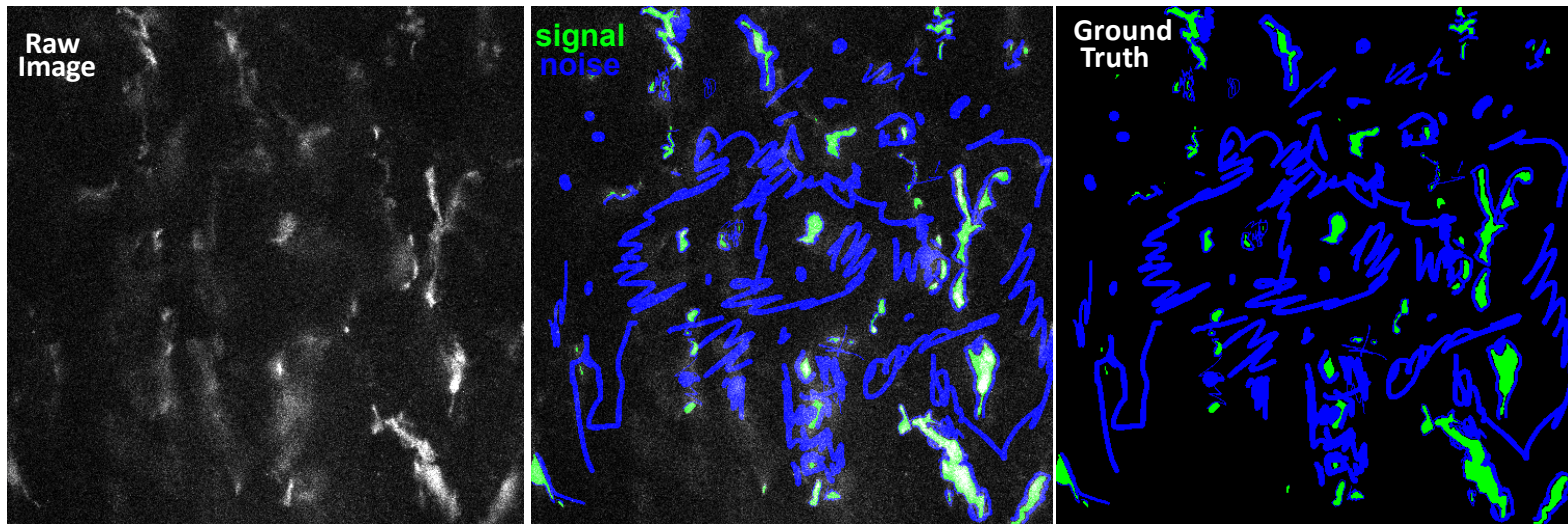**B**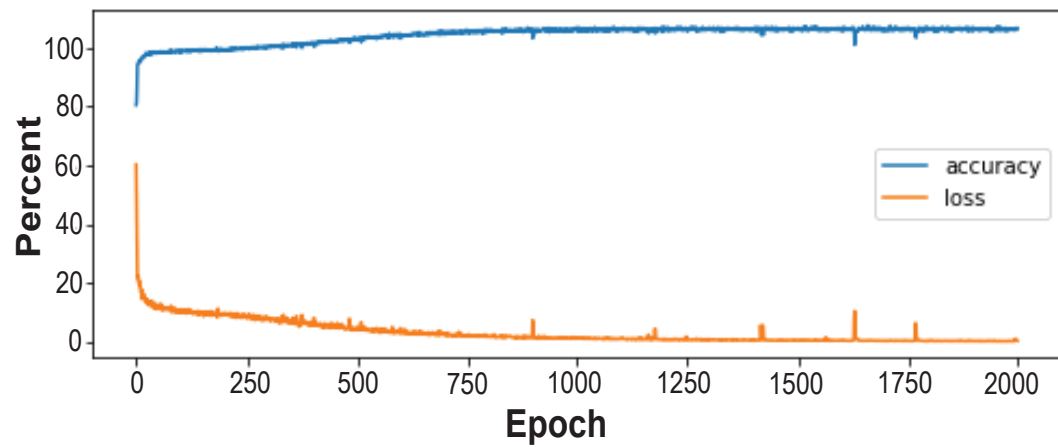**C**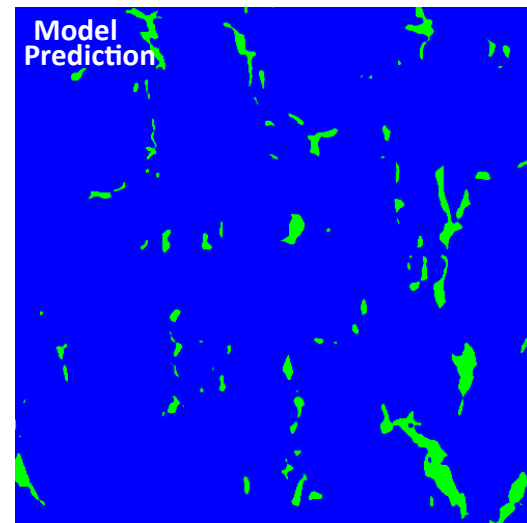

Supplement: Figure S2 [file NIHMS1792468-supplement-Figure_S2.pdf]

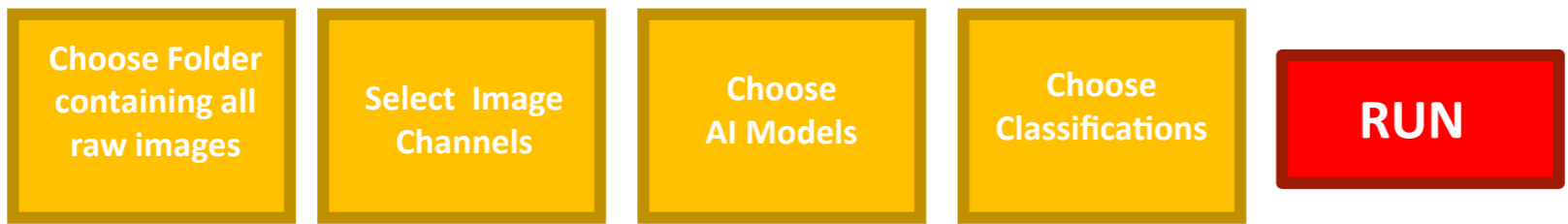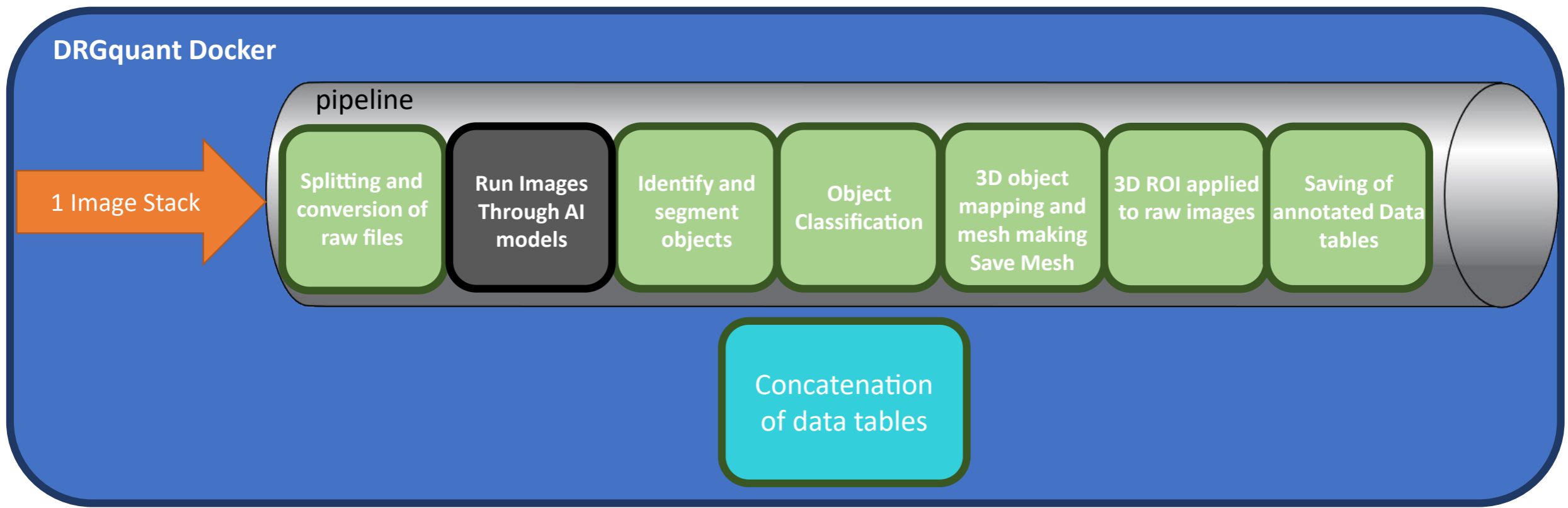

Supplement: Figure S3 [file NIHMS1792468-supplement-Figure_S3.pdf]
